# Pro12Ala polymorphism of peroxisome proliferator activated receptor gamma 2 may be associated with adverse neurodevelopment in European preterm babies

**DOI:** 10.1002/brb3.2256

**Published:** 2021-06-21

**Authors:** Suresh Victor, Andrew Chew, Shona Falconer

**Affiliations:** ^1^ Department of Perinatal Imaging and Health Centre for the Developing Brain School of Biomedical Engineering and Imaging Sciences King's College London London UK

**Keywords:** brain, cognition, genetic, infant, newborn, PPAR gamma, single‐nucleotide polymorphism

## Abstract

**Introduction:**

Prematurity is the leading cause of death and disability in children under 5 years of age. Understanding the molecular mechanisms of the biological processes involved in preterm brain injury may help develop novel neuroprotective treatment strategies. A growing body of evidence suggest that peroxisome proliferator‐activated receptor gamma (PPARγ) signaling is associated with inhibited brain development in preterm babies. The Ala allele of the Pro12Ala polymorphism of *PPARγ2* decreases receptor binding affinity and consequently induces a reduction of PPARγ signaling.

**Methods:**

In this study, we carried out a preliminary analysis of existing datasets to test the hypothesis that reduced transactivation capacity of PPARγ in the presence of the Ala variant of *PPARγ2* may be associated with adverse neurodevelopment in preterm babies.

The association between *PPAR‐γ2* Pro12Ala polymorphism and neurodevelopment at 18–24 months of age was assessed in two groups of European infants, 155 born before 33 weeks’ gestation and 180 born later than 36 weeks’ gestation using a linear regression model. The Bayley Scales of Infant and Toddler Development–3rd edition was administered to assess neurodevelopment at 18–24 months of age.

**Results:**

We observed the Ala allele of the Pro12Ala polymorphism in 25% preterm infants and 20% term infants. The Ala allele of *PPARγ2* was significantly associated with adverse cognitive (*p* = .019), language (*p *= .03), and motor development (p = 0.036) at 18–24 months of age after taking into consideration the duration of ventilation, gender, and index of multiple deprivation scores, but without correction for potential shared ancestry. There was no association between the *PPAR‐γ2* Pro12Ala polymorphism and neurodevelopment in term infants.

**Conclusions:**

These preliminary data suggest that PPARγ signaling in the presence of the Ala variant of *PPARγ2* may be associated with adverse neurodevelopment in preterm infants suggesting that further studies are warranted.

## Abstract 2

There are multiple definitions of hyposmia in Parkinson's disease (PD), and subtle differences between them can lead to different and often non‐comparable results. To illustrate the need for a standardised definition of hyposmia in PD, that would adjust for age and gender and account for the background decline in olfactory performance with ageing, this paper compares four published definitions of hyposmia, demonstrating the effect of each definition on the research outcomes when applied to the same cohort.

## 1 INTRODUCTION

Premature birth is the commonest cause of death and disability in children under 5 years of age (Moore et al., 2013). Over 30% of survivors experience neurocognitive problems from early life lasting into adulthood, including anxiety, inattention, social‐communication problems, and socioemotional problems. Prematurity leads to the loss of 75 million disability adjusted life years per annum worldwide (Kassebaum et al., 2016). Preterm brain injury is multifactorial, with inflammation and hypoxic‐ischemia being the leading causes. The underlying molecular and biological mechanisms of preterm brain injury are complex and not fully understood.

Peroxisome proliferator‐activated receptor gamma (PPARγ) signaling has previously been associated with altered brain development in preterm infants (Boardman et al., 2014; Krishnan et al., 2016), and replicated this observation using an unbiased, machine learning strategy to interrogate magnetic resonance tractography and genome‐wide single nucleotide polymorphism (SNP) datasets (Krishnan et al., 2017). This SNP‐based genome‐wide imaging genomics analysis used sparse reduced‐rank regression with genome‐wide SNP genotyping and a probabilistic tractography phenotype (Krishnan et al., 2017). An association was detected between SNPs in *PPARγ* gene (rs17036282, rs6801982, rs4135334, rs4135336, rs4135342, and rs6442313) and inhibited brain development after adjusting for gestational age, post menstrual age, and ancestry. SNPs in *PPARγ* gene were significantly overrepresented among the variables with the highest ranking in the model, contributing to a broader significant enrichment of lipid‐related genes among the top 100 ranked SNPs (Krishnan et al., 2017). However, as none of these linked SNPs are clearly linked to functional effects, and while changes in diffusion magnetic resonance imaging (MRI) are associated with cognitive outcomes, there is considerable variance in the relationship. It therefore remained uncertain if the observed genetic‐imaging linkage represented a biological effect. Equally, as no previous data were available on term‐born infants, it was unclear if this effect was specific to preterm infants, although the role of PPAR*γ* in the inflammatory process, and the well‐established association between inflammation and adverse outcome after preterm birth suggested this was likely.

The most analyzed common functional variant in the *PPARγ* gene is the Pro12Ala polymorphism on exon B of the *PPAR‐γ2* (rs1801282), defined by a CCA to GCA mutation on codon 12 changing a proline (Pro) to an alanine (Ala). The Ala variant is found more frequently in individuals with European ancestry (10%−22%), less frequently in African (4.7%), and Asian ancestry (5%) and rare in Latin Americans (Sarhangi et al., 2020). Functional analysis showed that the Ala allele decreases the receptor binding affinity to the responsive elements and consequently inducing a reduction in transcriptional activity of PPARγ signaling (Deeb et al., 1998). Studying this functional variant of the *PPARγ* gene may therefore provide an insight into the role of PPARγ signaling in the outcomes of prematurity.

In this study, we aimed to obtain preliminary data to determine whether further large studies of the role of *PPARγ2* in neurodevelopment after preterm birth are likely to be fruitful. We combined European infants from two genotyped cohorts of preterm and term‐born infants to address the question of whether the Ala variant of *PPARγ2* is associated with adverse neurodevelopment in preterm but not term‐born infants.

## 2 METHODS

*Subjects*: Preterm infants born before 33 weeks’ gestation were recruited as part of the Evaluation of Magnetic Resonance Imaging to Predict Neurodevelopmental Impairment in Preterm Infants (ePRIME) study and the developing Human Connectome Project (dHCP), details of which have been published elsewhere (Edwards et al., 2018; Makropoulos et al., 2018). Babies born after 36 weeks’ gestational age were recruited as part of the dHCP. Both studies were conducted according to the principles of the Declaration of Helsinki, and ethical approval was obtained from the UK National Research Ethics Service. Written parental informed consent was obtained for all subjects.

*Phenotyping*: Gestational age was determined using fetal ultrasound measurement. In preterm babies, chronic lung disease was defined as oxygen requirement at 36 weeks’ corrected gestational age. Necrotizing enterocolitis (NEC) was diagnosed at surgery. Discharge weight *z* scores were calculated using Fenton 2013 Growth Calculator for Preterm Infants (Chou et al., 2020). Index for Multiple Deprivation quintile group were based on the Office of National Statistic Indices of Multiple Deprivation 2010 and was calculated using NPEU IMD tool (https://tools.npeu.ox.ac.uk/imd/).

Only preterm infants were admitted to intensive care. Duration of ventilation, duration of total parenteral nutrition, and discharge weight *z* scores were therefore calculated only for preterm babies.

All available neuroimaging (cranial ultrasound and/or MRI brain) were reviewed for extensive brain abnormalities, major focal destructive parenchymal lesions, multiple punctate white‐matter lesions, or white‐matter cysts that may have an impact on neurodevelopment.

*Genotyping*: Saliva samples were collected using Oragene DNA OG‐250 kits (DNAGenotek Inc., Kanata, Canada) and genotyped on Illumina Human OmniExpress‐24 v1.1 arrays (Illumina, San Diego, CA, USA) for ePRIME and Illumina Infinium Omni5‐4 arrays (Illumina, San Diego, CA, USA) for the dHCP cohort.

*Population stratification*: Information on self‐reported ethnicity (as defined in ISB Standard DSCN 11/2008) was collected by asking mothers (and fathers when present) to define themselves according to a list of options. The terms were drawn from Ethnic Category National Codes as in Department of Health Guidance at the time. Parental self‐reported ethnicity was summarized into broader categories. Our full sample includes infants from three different cohorts: European, Asian, and African according to the classification that is provided in a study by Morales et al. (2018). Only data from the European cohort were analyzed due to the high prevalence of the Ala allele in this group. Principal component analysis for ancestry was not included in the analysis.

*Follow‐up assessment*: The Bayley Scales of Infant and Toddler Development−3rd edition (Bayley‐III) was administered to assess neurodevelopment at 18–24 months (corrected for prematurity) by two psychologists unaware of the *PPAR‐γ2* Pro12Ala polymorphism. Three directly assessed scales were measured: cognitive, language, and motor development. The language scale explores two branches of the development, the receptive and expressive language. The motor scale permits the examination of both fine and gross motor skills. The scaled score and composite score were calculated for each scale and were adjusted for each child and age (days), using the correction manual tables.

*Sample size calculation*: Assuming a pooled standard deviation of 14, the study would require a sample size of 37 for the Ala polymorph group and 111 for the wild‐type group (i.e., a total sample size of 148; to ensure that the wild‐type group is three times larger than the test group) to achieve a power of 85% and a level of significance of 5% (two sided), for detecting a true difference in means of motor composite score on BSID III at 18 months of age between the polymorph and the wild‐type group of −8 (i.e., 89–97).

*Statistical analysis*: The effect of *PPARγ2* Pro12Ala polymorphism on neurodevelopment (motor, cognitive, and language composite scores) was determined under an additive genetic model using stepwise linear regression adjusted for gestational age, gender, and index of multiple deprivation in term infants. In preterm infants, the effect of *PPARγ2* Pro12Ala polymorphism on neurodevelopment (motor, cognitive, and language composite scores) was determined using stepwise linear regression adjusted for gestational age, gender, duration of total parenteral nutrition, duration of ventilation, index of multiple deprivation, and study cohort (ePRIME or dHCP) without correction for ancestry. The Ala allele was considered the effect allele. All regressions were tested for homoscedasticity, normality of the error distribution and multicollinearity. Statistical significance was determined at *p*‐value < .05. All statistical analyses were performed using SPSS software (version 26.0).

## 3 RESULTS

180 European infants born after 36 weeks’ gestation were identified in the dHCP. A total of 155 European infants born before 33 weeks’ gestation were identified from ePRIME (*n* = 131) and dHCP (*n* = 24). All infants were genotyped and completed 18–24 months neurodevelopment follow‐up

Tables 1 and 2 show the characteristics of the preterm and term cohorts, respectively. In the preterm group, the Ala12Ala group tended to have lower birth weights, but this did not reach statistical significance. However, by the time of discharge, these trends in bodyweight did not persist. The incidences of chronic lung disease and NEC were within anticipated frequencies for this cohort across> groups.

**TABLE 1:** Characteristics of the studied cohort of preterm infants

Characteristics	Pro12Pro (*n* = 116)	Pro12Ala (*n* = 35)	Ala12Ala (*n* = 4)
Male/female (*n*)	64/52	19/16	1/3
Gestational age at birth (w)	29.6 (2.3)	29.3 (2.4)	30.3 (2)
Birth weight (g)	1313 (399)	1327 (372)	1223 (455)
Birth weight *z* score	−0.09 (0.93)	0.22 (0.74)	−0.77 (0.92)
Duration of ventilation (d)	3.5 (9.2)	3 (5)	6.8 (12)
Duration of TPN (d)	11.7 (22.3)	10 (10)	9 (6.6)
Discharge weight (g)	2137 (552)	2129 (434)	2126 (497)
Discharge weight *z* score	−1.6 (0.94)	−1.7 (0.76)	−1.7 (0.88)
Diagnosis of chronic lung disease (*n*)	28 (24%)	10 (28.6%)	1 (25%)
Diagnosis of NEC at surgery (*n*)	3 (2.6%)	2 (5.7%)	0
Index of multiple deprivation	17.3 (11.1)	15.9 (10.4)	15 (6.8)
Corrected age at BSID (m)	19.8 (1.1)	19.7 (0.9)	20 (0.8)
Cognitive composite score	97.8 (12.8)	94.4 (12.9)	88.8 (8.5)
Language composite score	97.4 (16.9)	92 (18.9)	92 (14.1)
Receptive language scale	10 (3.2)	9 (3.5)	9.25 (3)
Expressive language scale	9.1 (3)	8.2 (3.4)	8 (2.1)
Motor composite score	97.3 (11)	95.8 (13)	86.5 (6.3)
Fine motor scale	10.8 (2.4)	10.5 (2.9)	9 (1.4)
Gross motor scale	8.2 (2)	8 (2)	6.5 (3.4)

Abbreviation: BSID, bayley scales of infant and toddler development; NEC, necrotizing enterocolitis; TPN, total parenteral nutrition

**TABLE 2:** Characteristics of the studied cohort of term infants

Characteristics	Pro12Pro (*n* = 143)	Pro12Ala (*n* = 34)	Ala12Ala (*n* = 3)
Male/female (*n*)	74/69	19/15	1/2
Gestational age at birth (w)	40 (1.33)	39.7 (1.3)	39.9 (2.7)
Birth weight (g)	3441 (525)	3450 (412)	2870 (1032)
Index of multiple deprivation	20.6 (13.4)	21.5 (15.3)	14.6 (1.8)
Chronological age at BSID	18 (2)	17.7 (3.2)	18 (0)
Cognitive composite score	102 (13)	101 (20.3)	97.5 (3.5)
Language composite score	100 (16)	103 (21)	100 (8.5)
Receptive language scale	10.8 (2.9)	11.7 (3)	12 (0)
Expressive language scale	9.37 (2.5)	9.7 (2.4)	8 (2.8)
Motor composite score	102 (12.9)	99.9 (19.1)	88 (16.9)
Fine motor scale	11.6 (2.2)	11.1 (2.7)	10.5 (0.7)
Gross motor scale	9 (2.2)	9.2 (2.3)	5.5 (6.4)

Abbreviation: BSID, bayley scales of infant and toddler development

All term infants had a MRI brain scan. Of the 155 preterm infants, only 125 had MRI brain scan at term corrected gestation, the remaining 30 had cranial ultrasound scans. None of the infants had extensive brain abnormalities, major focal destructive parenchymal lesions, multiple punctate white‐matter lesions, or white‐matter cysts.

Mean composite cognitive, language, and motor development scores were lower with the Ala allele in preterm babies. Statistically significant regression models including Pro12Ala polymorphism of *PPARγ2* are shown in Table 3.

**TABLE 3:** Interactions between duration of ventilation, index of multiple deprivation, gender, and *PPARγ2* Ala allele with neurodevelopment in the studied cohort of preterm infants

Model	Unstandardized B ± SE	Standardized beta	*p* Value	*R* ^2^	*p* Value
Cognitive composite score					
Duration of ventilation	−0.35 ± 0.113	−0.231	.002	0.167	.000
Index of multiple deprivation	−0.28 ± 0.089	−0.236	.002		
Gender	5.3 ± 1.945	0.206	.007		
*PPARγ2* Ala allele	−4.58 ± 1.925	−0.179	.019		
Language composite score					
Index of multiple deprivation	−0.452 ± 0.123	−0.283	.000	0.129	.000
Gender	7.36 ± 2.67	0.212	.007		
*PPARγ2* Ala allele	−5.806 ± 2.643	−0.168	.03		
Motor composite score					
Duration of ventilation	−0.424 ± 0.1	−0.313	.000	0.194	.000
Gender	4.78 ± 1.7	0.207	.006		
Index of multiple deprivation	−0.215 ± 0.08	−0.202	0.007		
*PPARγ2* Ala allele	−3.6 ± 1.7	−0.157	.036		

There were no statistically significant models predicting neurodevelopment in term babies. Mean motor composite score was lower in Ala12Ala group, but this did not reach statistical significance (Table 2).

## 4 DISCUSSION

The mechanisms of abnormal brain development in preterm infants are largely unknown, but are thought to involve complex metabolic and inflammatory processes. PPARγ is a ligand activated transcription factor involved in the regulation of adipogenesis, glucose and lipid homeostasis, insulin sensitivity (Fuentes et al., 2013), and in the modulation of inflammatory responses (Croasdell et al., 2015). Previous studies have shown an association between PPARγ and brain microstructural development after preterm birth. The present study provides preliminary evidence of an association between PPARγ signaling and neurocognitive outcome in preterm infants. These results suggest that further studies in larger cohorts that allow better control for ethnicity and ancestry may help elucidate the mechanisms of abnormal brain development after preterm birth.

The Ala allele prevalence was slightly higher in preterm infants (25%) than in term infants (20%). The higher incidence in preterm babies is consistent with other studies and indicates a possible role of Pro12Ala polymorphism of *PPARγ2* in preterm birth. Meirhaeghe et al. (2007) also showed that the Ala variant occurs more frequently in individuals born preterm (35%, *n* = 60) than in individuals born at term gestation (22%, *n* = 942) and increases further in those born before 35 weeks’ gestation (50%, *n* = 24).

In term infants, the Pro12Ala polymorphism of *PPARγ2* was not associated with changes in neurodevelopment at two years of age. Although mean motor development scores of the Ala12Ala polymorphism were poorer, there were only two babies in this group and only one of two had adverse neurodevelopment. In contrast, Pro12Ala polymorphism of *PPARγ2* was associated with adverse neurodevelopment in preterm babies. In an observational study of 138 mother–infant pairs, Torres‐Espínola et al. (2015) showed that 18 months old term infants born to mothers with wild‐type Pro allele had better cognitive (Odd ratios (OR) = 5.11, 95% CI: 1.379−18.96, *p* = .015), language (OR = 3.41, 95% CI: 1.35−11.24, *p* = .044), and motor development scores (OR = 4.77, 95% CI: 1.243−18.33, *p* = .023) than the Ala allele carriers. The term infant's own genotype showed no relationship to neurodevelopment. They speculated that the maternal *PPARγ* may offer a transplacental protection to the term born fetal brain. Indeed, PPARγ is strongly expressed in human placenta (Abbott, 2009). Activation of PPARγ in placenta enhances the uptake of fatty acids necessary for fetal brain development and alters the expression of several proteins associated with fatty acid transport (Schaiff et al., 2007). This protection offered by the maternal genotype may be lost when the foetus is delivered prematurely.

Preterm delivery is associated with elevated risks of metabolic syndrome (dyslipidemia, insulin resistance, and hypertension), type 2 diabetes, and cardiovascular disease in adulthood (Barker et al., 1989; Hales et al., 1991; Rich‐Edwards et al., 1999). The association of PPARγ2 Pro12Ala polymorphism is well established in type 2 diabetes. A large‐scale association study of type 2 diabetes cases and controls predominantly of European ancestry confirmed that the Pro allele was associated with 13% increased risk of type 2 diabetes (Morris et al., 2012). A meta‐analysis of subjects from diverse ethnic backgrounds demonstrated that the Pro allele was associated with a lower body mass index. There was a trend for a stronger effect of the Pro allele in individuals of European ancestry (Galbete et al., 2013). PPARγ may therefore provide a link between prematurity and problems in adulthood.

Evidence suggests that PPARγ signaling is underactive in preterm infants during the newborn period. *PPARγ* expression has been demonstrated across the human and mouse brain and elevation in PPARγ expression has been noted through development until term equivalent age (Krishnan et al., 2017). Consistent with these data is the blood concentrations of adiponectin in preterm babies during the neonatal period. Adiponectin production is tightly regulated by *PPARγ*, and its blood concentrations represent a robust biomarker of *PPARγ* activity (Wagner et al., 2009). Two observations are relevant in relation to blood adiponectin concentrations and by implication *PPARγ* activity in preterm infants, (1) extreme preterm babies have much lower concentrations (19.9 ± 10.6 g/ml) than term babies (33.7 ± 13.6 g/ml) (Kajantie et al., 2004) and (2) concentrations increase during the first two to three weeks of age, but this increase is not sustained resulting in lower concentrations at term equivalence in comparison to healthy term infants (Hansen‐Pupp et al., 2015).

A substantial body of evidence demonstrate the role of PPARγ agonists in neuroprotection. Pioglitazone, a PPARγ agonist, acts as a repressor of two signaling pathways associated with preterm brain injury: (1) NF‐kappa B signaling, which promotes inflammation mediated injury (Kaplan et al., 2014), and (2) WNT canonical pathway, which inhibits developmental myelination (Vallée et al., 2018). In response to inflammatory stimuli, PPARγ agonists promote substantial upregulation of PPARγ expression in microglia (Drew et al., 2015) and normalize microglial response. PPARγ agonists induce axonal growth, attenuate mitochondrial dysfunction, and promote the differentiation of oligodendrocyte precursors into mature oligodendrocytes (Bernardo et al., 2009).

We were not surprised to find the duration of ventilation, socio‐economic deprivation, and gender to be strong predictors of adverse neurodevelopment in preterm babies; others have found similar predictors (Crilly et al., 2020). Although PPARγ participates in the control of inflammation, we did not find any increase in the prevalence of inflammation‐associated disorders of prematurity such as chronic lung disease and necrotizing enterocolitis in association with variants of *PPARγ2*. This may be because the etiology of these disorders is multifactorial and inflammation is only one of the many risk factors (Gephart et al., 2012; Trembath & Laughon, 2012).

Our results cannot be generalized to all preterm infants. Our cohorts did not include infants with significant brain injury or severe comorbidities and death. We also chose to study preterm infants with only European infants as the Pro12Ala polymorphism is found most commonly in this ethnic group. Further, large‐scale studies of different *PPARγ* variants are required in other ethnic groups to evaluate the relative impact of variation in PPARγ signaling on the morbidity and mortality of preterm infants. The availability of agonists for PPARγ signaling allows the exploration of a repurposing strategy to test this approach in the clinic.

## AUTHOR CONTRIBUTIONS

Suresh Victor was involved in study design, data interpretation, statistical analysis, and drafting the manuscript. Andrew Chew and Shona Falconer were involved in data acquisition and interpretation, and reviewing the manuscript.

### PEER REVIEW

The peer review history for this article is available at https://publons.com/publon/10.1002/brb3.2256.

## CONFLICT OF INTEREST

The authors declare no conflict of interest.

## Data Availability

The data that support the findings of this study are available from the corresponding author upon reasonable request.
